# Leveraging patient‐reported outcomes (PROs) in patients with pancreatic cancer: The Pancreatic Cancer Action Network (PanCAN) online patient registry experience

**DOI:** 10.1002/cam4.4257

**Published:** 2021-09-03

**Authors:** Arjun Gupta, Omar Khalid, Cassadie Moravek, Anica Lamkin, Lynn M. Matrisian, Sudheer Doss, Crystal S. Denlinger, Andrew L. Coveler, Colin D. Weekes, Eric J. Roeland, Andrew E. Hendifar, Ryan D. Nipp

**Affiliations:** ^1^ University of Minnesota Minneapolis Minnesota USA; ^2^ Pancreatic Cancer Action Network Manhattan Beach California USA; ^3^ Fox Chase Cancer Center Philadelphia Pennsylvania USA; ^4^ Seattle Cancer Care Alliance/University of Washington Seattle Washington USA; ^5^ Massachusetts General Hospital and Harvard Medical School Boston Massachusetts USA; ^6^ Samuel Oschin Cancer Center Cedars‐Sinai Medical Center Los Angeles California USA

**Keywords:** PanCAN, pancreatic cancer, patient‐reported outcomes, registry

## Abstract

**Background:**

The Pancreatic Cancer Action Network (PanCAN) Patient Registry is an online, pancreatic cancer‐specific, global registry enabling patients to self‐report sociodemographics, disease/management characteristics, and patient‐reported outcomes (PROs). We sought to describe the creation, user experience, and research potential of the PanCAN Registry.

**Methods:**

We obtained data to describe (1) the creation of the Registry (questionnaire development, marketing efforts, and regulatory considerations); (2) the user experience (user characteristics and interactions with the registry following inception); and (3) the research potential of the registry (comparing PROs and treatment patterns by age [±65 years] and treatment site [community or academic] for users with de novo metastatic disease).

**Results:**

The Registry was conceived as part of PanCAN’s strategic plan for a personalized therapy initiative. PanCAN staff and disease expert consultants developed questionnaires hosted on an electronic PRO platform. Users had the option to include their data in research efforts, and the Registry platform received institutional review board approval. From 7/2015 to 12/2020, 2187 patients visited the registry and 1697 (77.6%) completed at least one survey (median age = 64 years [range: 24–90], 47.9% women, 88.7% White, 34.0% metastatic disease). Among patients with metastatic disease (*N* = 567), 46.0% were ≥65 years old and 67.5% received treatment at community sites. Patients ≥65 years reported feeling less hopeful about the treatment plan (12.4% vs. 24.3%, *p* = 0.003), and patients treated at community sites reported more frequent treatment breaks of >2 weeks (58.2% vs. 28.1%, *p* < 0.001).

**Conclusions:**

Our findings demonstrate the feasibility, usability, and research potential of an online PRO registry for patients with cancer. This description of the PanCAN Registry should inform future registry‐building efforts to facilitate standardized PRO reporting and provide a valuable research database.

**Clinical** **trial registration number:** Not applicable.

## Graphical abstract

The Pancreatic Cancer Action Network (PanCAN) Patient Registry is a voluntary, global, pancreatic cancer‐specific registry enabling patients to self‐report sociodemographics, disease/ management characteristics, and patient‐reported outcomes. In this manuscript, based on over 2000 patients visiting the PanCAN Registry over a 5‐year period, we describe its creation, user experience, and research potential, which can guide future registry building efforts (including standardized patient‐reported outcomes collection).

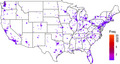

## 1 INTRODUCTION

Pancreatic cancer is a highly lethal cancer and one of few cancers with a rising incidence and mortality in the United States.^1^ The American Cancer Society estimates that in 2021 over 60,000 people will be diagnosed with pancreatic cancer, and over 48,000 will die from this cancer.^1^ Most patients are diagnosed at an advanced stage of disease, resulting in poor survival (5‐year survival of approximately 10%).^2^ Even for patients diagnosed with early‐stage disease, 5‐year survival remains limited (approximately 20%).^2^ Notably, these poor survival outcomes are accompanied by a significant symptom burden related to the cancer and its treatment, which impacts patients’ quality of life and use of health‐care services.^2^, ^3^

Patient‐reported outcomes (PROs) assessing important patient‐centered issues, such as symptoms and quality of life, are increasingly incorporated into cancer care to enhance patients’ care experience and outcomes.^4^, ^5^, ^6^ The incorporation of PROs, followed by appropriate clinical follow‐up, has demonstrated the potential to improve patients’ symptoms, quality of life, health‐care use, and even survival.^4^, ^7^ PROs may also provide prognostic and predictive information in patients with advanced cancer.^3^ As PROs become more commonly integrated into routine care across health systems, a growing need exists to understand if PROs can be broadly implemented and collected from patients across geographic areas.

An online PRO registry, collecting electronic PROs from patients and caregivers across the globe for a single disease process, could have a tremendous impact on patients. However, a dearth of research exists regarding such efforts. Therefore, a description of the steps required to create such a registry could serve as a reference for future registry efforts across diverse diseases. Capturing PROs at scale, and collating and analyzing this information, could have important clinical, research, and policy implications by assessing patient needs and identifying patterns of care delivery and outcomes.^8^, ^9^ Large‐scale PRO collection represents a particularly important endeavor for a disease, such as pancreatic cancer, associated with a considerable symptom burden and high health‐care utilization.^3^, ^10^ The Pancreatic Cancer Action Network (PanCAN) Patient Registry represents one such initiative to develop an online, global PRO registry. The PanCAN Patient Registry is a pancreatic cancer‐specific online registry enabling patients worldwide to report sociodemographics, disease and management characteristics, and PROs via online surveys. In the current study, we sought to describe the creation, user (patient) experience, and research potential of the PanCAN Patient Registry (hereafter referred to as the *Registry*).

## 2 METHODS

This retrospective study aims to (1) outline the process of creating the Registry, (2) describe the associated user experience, and (3) highlight the Registry's research potential. The Registry platform received institutional review board (IRB) approval through the Western institutional review board via the Registry vendor. PanCAN updates the IRB every year via the Registry vendor to maintain the Registry and associated studies. When patients sign up for the Registry, they can set permissions for either PanCAN, PanCAN‐approved researchers, or all researchers to access de‐identified health data and identified contact information.

### 2.1 Creation of the Registry

To describe the creation of the Registry, we descriptively outline the vision for the Registry, the development of questionnaires (the PRO component), and information technology support required to build an electronic PRO platform with a user interface, regulatory requirements, and marketing efforts to recruit users.

### 2.2 User experience

To assess the user experience and rate of users joining the Registry, we assessed individual patient characteristics and interactions with the Registry (e.g., number of visits, survey completions, motivation for joining the Registry) for patients who provided permission to use their data. We included data collected from July 2015 (Registry inception) to December 2020. We analyzed these data descriptively.

### 2.3 Research potential

To evaluate the concordance between patient‐reported and physician‐reported information, we compared Registry data to data provided by patients’ treating oncologists available through PanCAN’s parallel effort, called Know Your Tumor^®^ (KYT).^11^ KYT provides a source of physician‐reported data for a subset of Registry users. Enrolled KYT participants signed a patient confidentiality waiver and patient coordinators from Perthera, Inc. received patient records from the treating oncologist. For individuals enrolled in both KYT and participating in the Registry, we compared two data points: (1) age at the time of diagnosis of pancreatic cancer (*n* = 79) and (2) first systemic drug therapy regimen (*n* = 71).

To investigate the research potential of the Registry, we explored a subset of the Registry. Specifically, among patients reporting de novo metastatic disease who joined the Registry between July 2015 and October 2019, we compared PROs and treatment patterns by patient age (±65 years) and treatment site (community or academic, defined as absence or presence of an associated teaching facility, as determined by authors). We compared categorical variables using Chi‐squared tests. The current manuscript provides examples of the data that can be derived from such a PRO Registry.

## 3 RESULTS

### 3.1 Creation of the Registry

#### Vision

PanCAN initially planned the registry in 2014 as part of the strategic plan for a personalized therapy initiative. A subset of PanCAN’s Scientific and Medical Advisory Board helped to serve as an advisory board for the personalized therapy initiative. This broader initiative included: (1) the KYT personalized therapy initiative and (2) the Registry. PanCAN designed the KYT initiative to provide biomarker testing to patients with pancreatic cancer.^11^, ^12^, ^13^ The Registry was envisioned with two purposes in mind: (1) to provide the patient perspective to augment the physician‐reported outcomes of the KYT initiative and (2) to provide an opportunity for those unable to enroll in KYT or a clinical trial to participate in research. KYT launched in June 2014. Beta testing of the Registry occurred from July 2015 to December 2015, with a formal public launch in January 2016.

#### Preparing, vetting, and uploading questionnaires/instruments

PanCAN chose to utilize the PEER (Platform for Engaging Everyone Responsibly) platform maintained by Genetic Alliance to host the Registry.^14^, ^15^ PanCAN staff, working with pancreatic cancer disease experts serving as consultants, developed the Registry questionnaires with input and review by members of the PanCAN Scientific and Medical Advisory Board. The Patient Services team also provided feedback and reviewed the surveys based on experiences with patients who contacted the PanCAN call center. Content developers knew that PROs would be collected online (vs. paper) and considered that while designing questionnaires. PanCAN staff worked with Genetic Alliance and their Information Technology vendor to build out the Registry platform surveys, including branching logic and longitudinal survey data collection, as required.

In total, users have the opportunity to complete 27 unique surveys on the Registry website. One of the surveys, named the “Basics survey,” includes information on patient characteristics (age at diagnosis, disease stage, etc.) and the motivation for joining the Registry, which lays the foundation for other surveys. Users must first take the Basics survey before they can take additional surveys. Thus, we defined users as Registry participants who had completed at least the Basics survey for this study. The Health Assessment survey derives from the Patient‐Reported Outcomes Measurement Information System (PROMIS)‐29 validated survey.^16^ Other surveys inquire about general demographic information, choices and site(s) of receiving care, diagnostics (laboratory‐based, imaging, genomic), treatments and associated adverse effects, and symptom management. We present the Basics survey in Table S1 and a list of all surveys in Table S2.

#### Legal and regulatory requirements

The Registry platform has IRB approval which is updated annually through Genetic Alliance. For research studies, a PanCAN staff member serves as the principal investigator and manages regulatory requirements. Users can set their preferences for who can access their data and whether their data can be used for research.

### 3.2 Connecting with patients, and marketing efforts

PanCAN utilized Patient Central (PanCAN’s patient‐facing portal that provides free, personalized information and resources to patients) to inform constituents contacting the call center about the Registry and worked with the marketing division to announce the launch of the Registry to recruit participants from the existing constituent database.^17^ The Registry was advertised as a place to collect and store all of the patient's medical records and data in one, easy‐to‐access location, and to share data with researchers to advance pancreatic cancer research. Additionally, patients who enrolled in the KYT program can complete surveys while waiting for and after receiving their genomic report through follow‐up by the KYT Manager and the Registry Manager.

**FIGURE 1:**
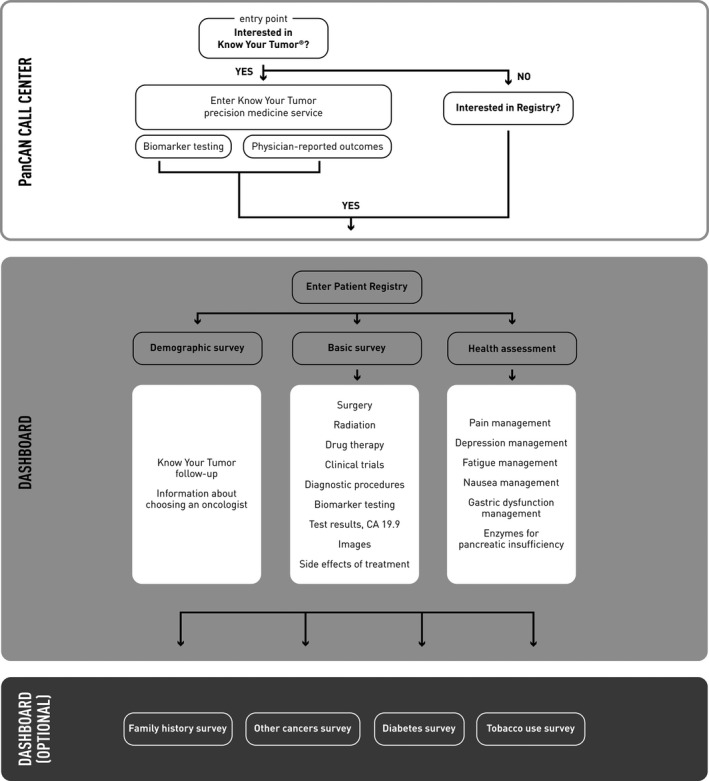
The workflow as it appears to PanCAN Registry users. Individuals enter the PanCAN Patient Registry from the PanCAN.org website or by contacting the Patient Central call center by phone or email (top box). Individuals who enroll in the Know Your Tumor personalized medicine service are offered the opportunity to enroll in the Patient Registry; others are directed to the Registry if they express interest. The Basic, Demographic, and Health assessment surveys are found on the dashboard after data access and privacy settings are selected (center box). The Basic survey must be completed first and Registry users are defined as those that completed the Basic survey. The answers to questions posed in the Basic and Health assessment surveys trigger the appearance of additional surveys on the dashboard if they are relevant for the participant (white boxes). Additional optional surveys appear on the dashboard at appropriate times to avoid overwhelming the participant (bottom box)

From a user's perspective, when individuals sign up for the Registry (website: https://www.pancan.org/facing‐pancreatic‐cancer/patient‐services/patient‐registry/) and complete the privacy settings, they are directed to the Basics survey. Depending on patients’ answers to specific questions, follow‐up surveys appear on their Dashboard with more detailed questions about diagnostics, treatments, and symptoms. Users may also answer questions on family history, other cancers, diabetes, and tobacco use. Figure 1 presents the workings of the dashboard and how surveys are introduced to Registry users.

### 3.3 User experience and rate of new users

**FIGURE 2:**
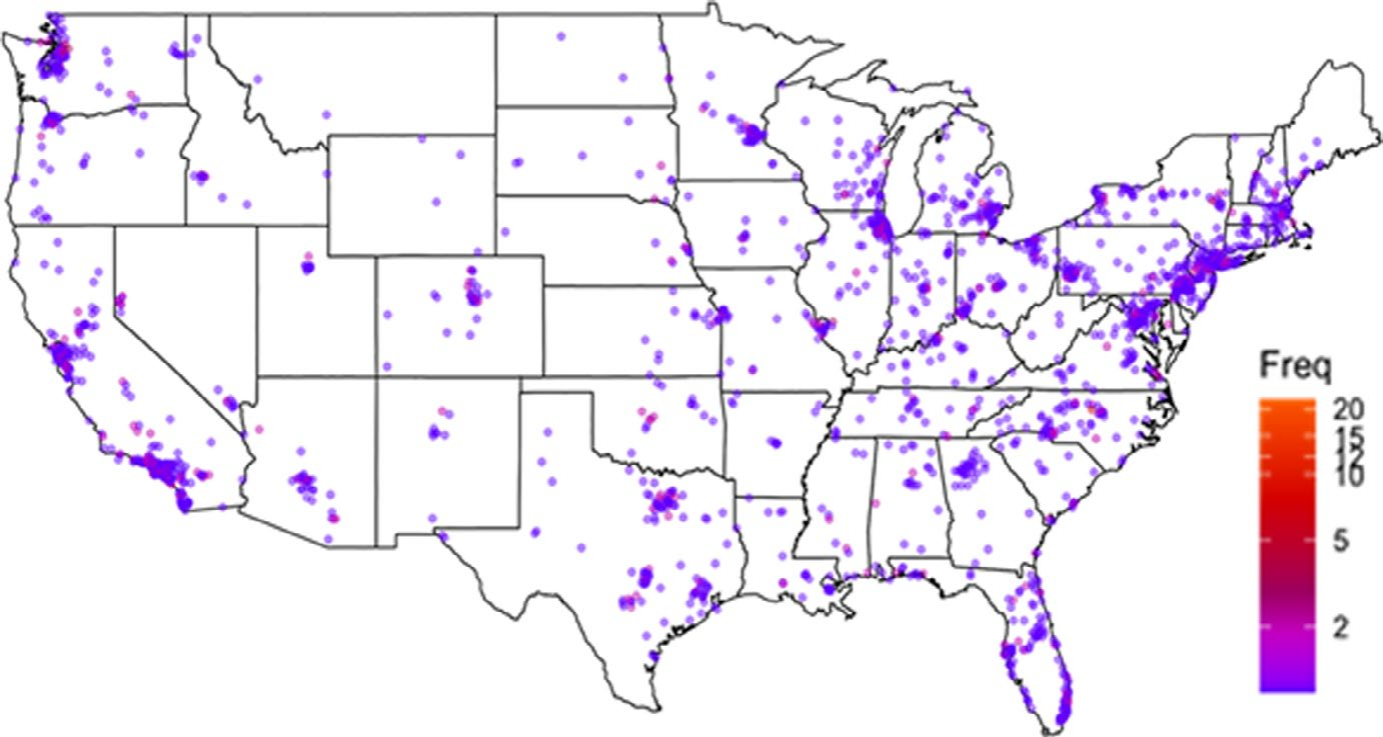
Heat map of users of the PanCAN Patient Registry within the United States

Of 2187 patients who visited the Registry and started the Basics survey from July 2015 to December 2020, 1697 (77.6%) completed the Basics survey, and were included in this analysis (median age = 64 years [range: 24–90], 47.9% women, 88.7% White). Less than 2% of users skipped reporting the stage of pancreatic cancer at diagnosis, and of those who reported the stage, 34.0% had metastatic disease. Common motivations for joining the Registry were to help collect information that could support pancreatic cancer research and other patients (95%) and to learn more about pancreatic cancer (90%). Complete patient demographics and characteristics are presented in Table 1. Users represented patients from all 50 United States and 28 countries. Figure 2 shows a heat map of Registry users within the United States. After the Basics survey (which by definition had a 100% completion rate), the most commonly completed surveys were the General Information (66%) and Drug Therapy (59%) surveys. There was an initial rapid uptake in users: in the first complete year after the launch (2016), 535 new users joined the Registry. These numbers declined annually, with 119 new users in 2020 (Table 1).

**TABLE 1:** Demographics and characteristics of patients in the PanCAN Registry^a^

Overall cohort	
Number completing ‘’Basic Survey’’ (this formed the baseline population of ‘’Users’’)	1697
Age, years, median (range)	64 (24–90)
Age, years	
≥65	835 (49.2%)
<65	861 (50.8%)
Sex	
Female	525 (47.7%)
Male	573 (52.1)
Neither	1 (0.1%)
Skipped	598
Gender	
Women	531 (47.9%)
Men	576 (52.1%)
Skipped	590
Race	
White	1072 (88.7%)
Hispanic, Latino, or Spanish origin	45 (3.7%)
Asian	36 (3.0%)
Black or African American	30 (2.5%)
American Indian or Alaskan Native	18 (1.5%)
Native Hawaiian or Other Pacific Islander	3 (0.2%)
Other	4 (0.3%)
Skipped	489
Year of sign‐up	
2015	25 (1.5%)
2016	535 (31.5%)
2017	436 (25.7%)
2018	306 (18.0%)
2019	276 (16.3%)
2020	119 (7.0%)
Stage of cancer at diagnosis	
Resectable	380 (22.7%)
Borderline resectable	409 (24.5%)
Locally advanced	244 (14.6%)
Metastatic	567 (34.0%)
I am not sure	69 (4.2%)
Skipped	28
Treatment site	
Community	482 (59.7%)
Academic	326 (40.3%)
Skipped	889
Reason for joining the Registry (multiple options allowed, percentage who strongly agree or agree)	
To provide information for researchers and other patients	95%
To learn more about pancreatic cancer	90%
To share information with friends, family, or a doctor	60%
To organize medical records	40%
Someone (e.g., family member, doctor) asked me to	31%
Rates of Common Surveys completed	
Basics survey	100%
General information	66%
Drug therapy	59%
Surgery	38%
For patients with initial diagnosis of metastatic cancer (*n* = 567)	
Age, years	
≥65	261 (46.0%)
<65	306 (54.0%)
Treatment site	
Community	187 (62.5%)
Academic	112 (37.5%)
Skipped	268

^a^ Data are presented as number (percentage) unless stated otherwise. ‘’Skipped’’ answers are not included while calculating percentages.

### 3.4 Research potential

We present some examples of the research potential of the Registry. When comparing patient‐reported and physician‐reported information on age at the time of diagnosis, the concordance was 94.9% (75/79). For situations in which there was discordance (*n* = 4), the age difference was within 2 years. For the first line of systemic chemotherapy (name of regimen), the concordance between patient‐reported and physician‐reported information was 88.7% (63/71). In all situations that we noted a discrepancy (*n* = 8), the first‐line chemotherapy regimen indicated by the patient was administered at some point in their clinical course, per physician‐reported information, but in a later line of therapy.

**FIGURE 3:**
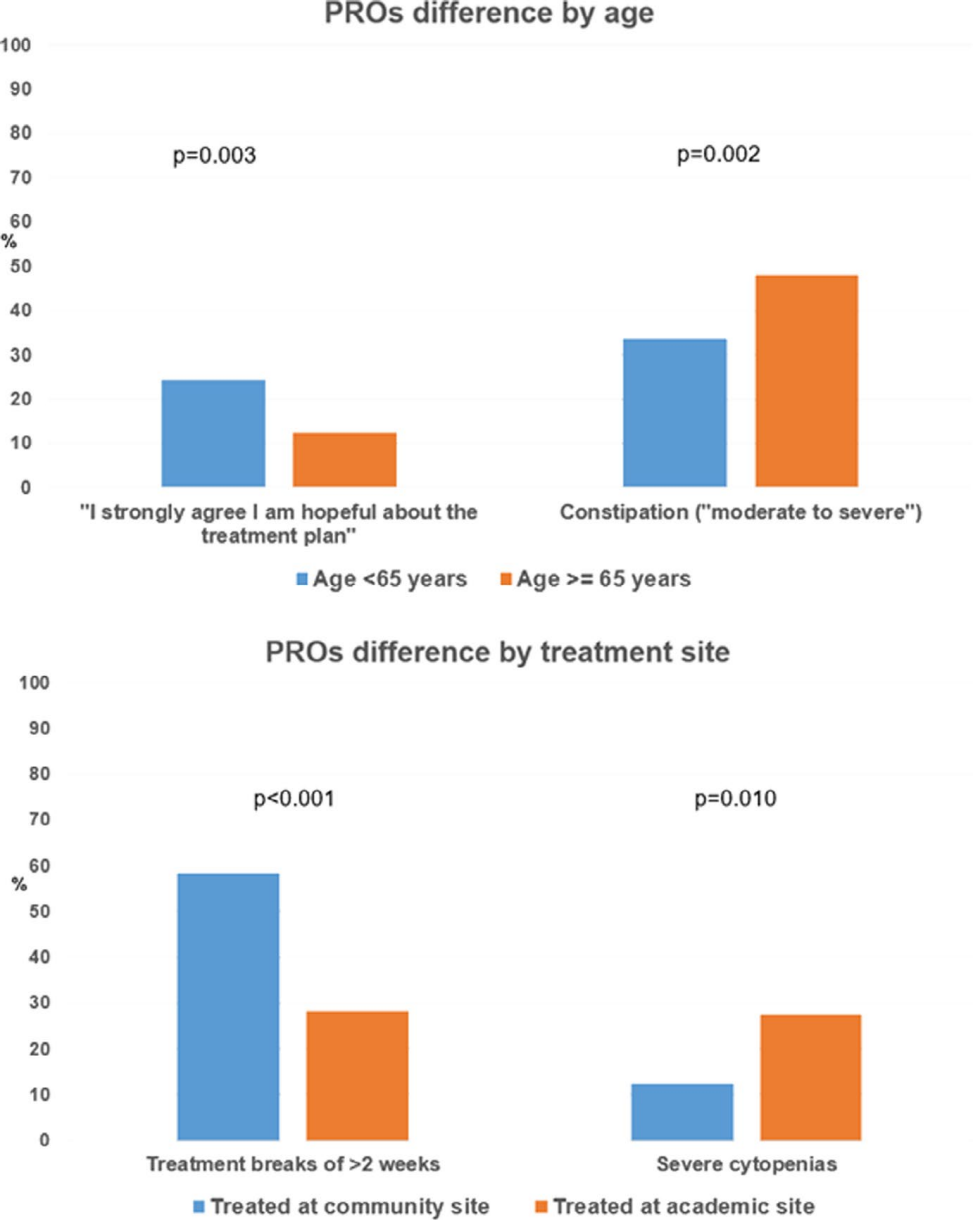
Differences in PROs in patients with metastatic pancreatic cancer based on age and treatment site

Among patients with de novo metastatic disease, 46.0% were aged 65+ and 62.5% received treatment at community sites (Table 1). Patients aged ≥65 years reported feeling less hopeful about the treatment plan (strongly agree: 12.4% vs. 24.3%, *p* = 0.003) and reported more constipation symptoms (moderate/severe: 47.9% vs. 33.6%, *p* = 0.002) compared with patients <65 years old (Figure 3). Patients treated at community sites reported more frequent treatment breaks of 2+ weeks (58.2% vs. 28.1%, *p* < 0.001) and less frequent severe cytopenias (12.4% vs. 27.4%, *p* = 0.010) compared with those treated at academic sites.

## 4 DISCUSSION

With over 2000 patients with pancreatic cancer visiting the PanCAN Registry from July 2015 to December 2020 and more than 75% completing a survey, our findings demonstrate the feasibility, usability, and research potential of a global, online, disease‐specific PRO registry. We noted high rates of voluntary engagement from patients across the United States and internationally, who largely participated to advance pancreatic cancer research. We observed important differences by age and treatment site regarding patients’ perceptions of their treatment plan, symptom burden, treatment patterns, and side effects. With an increasing focus on using PROs to enhance patient outcomes, online registries like the PanCAN Registry can help to facilitate standardized PRO reporting and monitoring while also providing a valuable research database.

Findings from this initial experience with the PanCAN Registry can inform registry building efforts for other cancers and health conditions by providing a methodology template and investment estimates. PanCAN envisioned and implemented this Registry, with two other groups serving as stakeholders: (1) pancreatic cancer disease experts who helped to develop questions; and (2) patients who voluntarily reported data. Future efforts must recognize that several stakeholders may be interested in building such a registry, with different but aligning motivations. Stakeholders for registries can include patients and advocacy organizations, investigators and academia (both researchers and clinicians), and industry and regulatory agencies.^18^ The global nature of this Registry might be particularly relevant to rarer conditions, where the ability to aggregate PRO data in a standardized manner could overcome the limitations of fewer data points if limited to a single institution and/or area.^18^ Other cancer site‐specific registries include those for relatively less common cancers, such as pediatric adrenocortical carcinoma, gastrointestinal stromal tumors, and angiosarcoma,^19^, ^20^, ^21^ and for more common cancers, such as lung cancer and metastatic breast cancer.^22^, ^23^ Much like the broader PanCAN mission to promote genetic testing of patients through the KYT program, some cancer registries, such as the International Pediatric Adrenocortical Tumor Registry, the Angiosarcoma Project, and the Metastatic Breast Cancer Project, allow patients to share biospecimens (germline and somatic) for genetic testing, along with clinical information.^20^, ^21^, ^22^ Analyses of clinical information allow research insights, such as identifying ‘’exceptional responders’’,^21^, ^22^ exploring patterns of care delivery and outcomes,^19^, ^20^, ^24^ and evaluating specific needs, such as those related to patient education resources.^23^ These registries are often supported by foundations and professional organizations, rely heavily on direct‐to‐patient outreach through social media, and promote a patient‐partnered approach to research. Thus, along with prior initiatives, we envision that this Registry effort can guide future actions, although every disease process and initiative will have a unique mission and needs.

We found high rates of user engagement with the Registry despite limited marketing efforts. The current Registry effort leveraged PanCAN’s existing constituent base, with the ability to direct patients to the Registry from the KYT program and those contacting PanCAN’s patient services contact center. Most users who signed up reported doing so for altruistic reasons, namely, to advance the pancreatic cancer research field and help other patients. As evidenced by high rates of completion (>75%) of the Basics survey among patients visiting the Registry, and use by patients across the age spectrum, including older adults (up to age 90), the electronic PRO platform provided a user‐friendly experience. However, several challenges merit discussion. First, we found limited racial diversity among users. In an analysis of racial disparities in treatment/outcomes of over 20,000 patients with pancreatic cancer between 1992 and 2011 in the Surveillance, Epidemiology, and End Results‐Medicare database, 83.9% were White and 9.0% were Black.^25^ In the United States Cancer Statistics database, from 2014 to 2018, 82.0% of pancreatic cancer cases occurred in Whites and 12.7% in Blacks.^26^ Thus, the 2.5% representation of Black patients in the current registry likely represents their under‐representation compared to the proportion of pancreatic cancer cases in Black individuals from other datasets. The COVID19 pandemic highlighted issues with racial disparities in telehealth access and use,^27^ and the use of an electronic patient portal may represent a barrier for some patients to participate. Additionally, although users represented all 50 states, our review of the specific locations highlighted that most of the patients resided on the coasts, with smaller numbers from the Midwest. Furthermore, we observed a consistent drop‐off in the number of new users engaging with the Registry per year, following 2016. This drop‐off may be explained by an initial influx of constituent members with prevalent disease, and then subsequent uptake in more recent years likely consisted of people with newly diagnosed, incident disease. The Registry invested little in external marketing efforts, and additional work is needed to understand how investments in marketing and outreach could help maintain active engagement with the portal. Thus, concerted efforts are required to understand racial and geographic barriers to participation, and to ensure (1) equitable access to PanCAN resources and (2) that existing and new users continue to find value with Registry participation. As part of a strategic overhaul from the next fiscal year, PanCAN will (1) engage in active outreach and marketing, especially to serve traditionally underserved and minority populations, and (2) re‐evaluate the PRO platform and existing questionnaires with a specific focus on augmenting long‐term data collection. We hope this targeted recruitment strategy will help underserved patients access PanCAN and its resources, and updated questionnaires will allow us to collect and analyze longitudinal data.

Data accuracy and research potential represent critical considerations in developing and managing a PRO database, particularly with online, unsupervised, and voluntary data reporting. We observed high rates of concordance between Registry data and physician‐provided data. PROs, as they relate to patients’ symptoms and quality of life, are increasingly being incorporated into research and clinical practice.^5^, ^28^ Our current findings suggest that patient‐reported data may be reliable beyond symptoms and extend to more objective disease and treatment factors. Patient‐reported data may be especially pertinent while collating information in situations without easy access to standardized, objective medical data (e.g., non‐integrated electronic medical record data or non‐uniform data reporting). Highlighting the Registry's research potential, we observed that older patients reported feeling less hopeful about the treatment plan and reported higher rates of constipation than younger patients. These differences underscore the burden of, and unique supportive care needs for, older adults with cancer.^29^ We also found that patients treated at community sites reported more frequent treatment breaks and experienced fewer cytopenias. These findings provide important insights into potential differences in practice patterns across sites, and if consistent, can help guide day‐to‐day clinical practice for oncologists.^30^ Whether these differences translate into differences in patient outcomes merits further study. Overall, our findings highlight how the PanCAN Registry represents a fertile ground for investigation, including identifying unmet needs.

The current study contains several limitations. First, as mentioned previously, the Registry had an overrepresentation of White race participants, which could exacerbate existing racial disparities in representation. Second, the current study lacks a longitudinal evaluation of PROs. Future efforts should explore if/how (1) users naturally report PROs longitudinally; and (2) the Registry can promote longitudinal PRO reporting using nudges or other strategies. Third, Registry data tend to under‐report certain treatment options,^31^ and we found relatively lower rates of survey completions for questionnaires regarding treatment received (drug therapy, 59%; surgery, 38%). In the future, efforts to understand treatment paradigms will need to develop strategies for increased completion of these questionnaires. Lastly, Registry surveys have not been modified since the initial Registry creation, and in 2021 PanCAN plans to review and update all existing questionnaires.

In conclusion, the current study demonstrates the feasibility, usability, and research potential of an online, global, voluntary pancreatic cancer‐specific registry. PanCAN’s vision and investment successfully created the Registry, with input on questionnaires provided by pancreatic cancer experts. Patients who reported their data acted as natural stakeholders, motivated by advancing research and helping others. These findings can serve as a template for future registry‐building efforts for other cancers and disease conditions. By engaging established and new constituents and developing a user‐friendly electronic PRO interface, the Registry had high rates of natural engagement without extensive spending on marketing. We observed important differences by age and treatment site regarding patients’ symptoms and treatment patterns. Our findings demonstrate that registries, such as this, can facilitate standardized PRO reporting and monitoring from patients worldwide and provide a valuable research database.

## CONFLICT OF INTEREST

None.

## ETHICAL APPROVAL STATEMENT

This study was conducted in accordance with the ethical principles of the Declaration of Helsinki and consistent with Good Clinical Practice guidelines. The Registry platform received institutional review board approval through the Western institutional review board via the Registry vendor. PanCAN updates the IRB every year via the Registry vendor to maintain the Registry and associated studies. All patients set permissions to allow access to their de‐identified health data.

## Supporting information

Table S1‐S2

## Data Availability

Data available on request from the authors. The data that support the findings of this study are available from the corresponding author upon reasonable request.
