# Supplementary material for: Leveraging patient‐reported outcomes (PROs) in patients with pancreatic cancer: The Pancreatic Cancer Action Network (PanCAN) online patient registry experience
Source: Cancer Med. 2021 Sep 3;10(20):7152–61. doi: 10.1002/cam4.4257 (PMC8525124; doi:10.1002/cam4.4257)
Supplement: Supplementary file 1 — Table S1‐S2 [file CAM4-10-7152-s001.docx]

**Supplementary Table 1: The Basics Survey**

- I was diagnosed with pancreatic cancer: yes/ no

Would you like to enter information for someone else who has been diagnosed with pancreatic cancer?

- Please let us know why you joined our Patient Registry:
- The first time I was told I had pancreatic cancer was: (timing)
- When I was first diagnosed with pancreatic cancer, my cancer was: (stage)
- Currently, my pancreatic cancer is: (stage)
- Currently, my pancreatic cancer is in the following organs:
- Before my diagnosis, I had the following symptoms:
- Before my diagnosis, I had symptoms for: (time frame)
- In the weeks following my diagnosis, I: (understanding, support)
- The type of pancreatic cancer I was diagnosed with: (ability to upload pathology report)
- I have received the following treatment(s): (modalities)
- Do you plan to receive treatments in the future?
- Are you currently on treatment?
- In the weeks following my diagnosis, I: (understanding of treatment plan)
- At any time since diagnosis, I have experienced the following: (symptoms)
- Have you ever taken digestive (pancreatic) enzymes?

**Supplementary Table 2: List of all Surveys Available to Registry Users.**

**General information**

Pancreatic Cancer Experience Basics

Health Assessment

General demographic information

**General Medical Health**

Diabetes

Family History

Other Cancers

Tobacco Use

**Care choices**

Clinical Trial

Information About Choosing an Oncologist

**Diagnostics**

CA19.9

Diagnosis and Procedures (ex: EUS, Biopsy, FNA)

Know Your Tumor Follow Up

Molecular Profiling

Scans

Test Results

Detailed Diagnosis

**Cancer Treatment**

Drug Therapy (ex: Chemotherapy, Targeted Therapy)

Radiation

Surgery

**Symptoms/ Management**

Depression Management

Digestion

Fatigue Management

Nausea Management

Pain Management

Pancreatic Enzymes
